# Improving the reversibility of thermal denaturation and catalytic efficiency of *Bacillus licheniformis* α-amylase through stabilizing a long loop in domain B

**DOI:** 10.1371/journal.pone.0173187

**Published:** 2017-03-02

**Authors:** Zhu Li, Xuguo Duan, Sheng Chen, Jing Wu

**Affiliations:** 1 State Key Laboratory of Food Science and Technology, Jiangnan University, Wuxi, China; 2 School of Biotechnology and Key Laboratory of Industrial Biotechnology, Ministry of Education, Jiangnan University, Wuxi, China; 3 Department of Food Science and Engineering, College of Light Industry Science and Engineering, Nanjing Forestry University, Nanjing, China; Russian Academy of Medical Sciences, RUSSIAN FEDERATION

## Abstract

The reversibility of thermal denaturation and catalytic efficiency of *Bacillus licheniformis* α-amylase were improved through site-directed mutagenesis. By using multiple sequence alignment and PoPMuSiC algorithm, Ser187 and Asn188, which located within a long loop in Domain B of *Bacillus licheniformis* α-amylase, were selected for mutation. In addition, Ala269, which is adjacent to Ser187 and Asn188, was also investigated. Seven mutants carrying the mutations S187D, N188T, N188S, A269K, A269K/S187D, S187D/N188T, and A269K/S187D/N188T were generated and characterized. The most thermostable mutant, A269K/S187D/N188T, exhibited a 9-fold improvement in half-life at 95°C and pH 5.5, compared with that of the wild-type enzyme. Mutant A269K/S187D/N188T also exhibited improved catalytic efficiency. The catalytic efficiency of mutant A269K/S187D/N188T reached 5.87×10^3^±0.17 g·L^-1^·s^-1^ at pH 5.5, which is 1.84-fold larger than the corresponding value determined for the wild-type enzyme. Furthermore, the structure analysis showed that immobilization of the loop containing Ser187 and Asn188 plays a significant role in developing the properties of *Bacillus licheniformis* α-amylase.

## Introduction

Alpha-amylases (1,4-α-glucan glucanohydrolase; EC 3.2.1.1), which catalyze the conversion of starch and related carbohydrates into sugar syrups of various types, are extensively utilized in the industries of detergent, textile, and food [1]. Among the α-amylases, *Bacillus licheniformis* α-amylase (BLA) is widely used in industrial starch hydrolysis processes for its better reversibility of thermal denaturation, compared with *Bacillus amyloliquefaciens* α-amylase (BAA) and *Bacillus stearothermophilus* α-amylase (BSTA) [2]. The differences in the reversibility of thermal denaturation of *Bacillus* α-amylases are quite interesting given the fact that the amino acid sequences [3] and three-dimensional structures of these three α-amylases are quite similar [4–6].

Three domains: A, B, and C constitute the structure of *Bacillus* α-amylases [7]. Domain A is the core of the α-amylase. It is composed of a central (β/α)_8_ barrel, which contains three catalytic residues: Asp231, Glu261, and Asp328 (BLA numbering). A sheet of four antiparallel β-strands and a pair of antiparallel β-strands separated by fairly long loops compose Domain B [8]. The Ca^2+^-Na^+^-Ca^2+^ binding site is located in the interior of domain B, spanning to the interface with domain A. Domain C forms a Greek key motif. It is located on the opposite side of the central (β/α)_8_ barrel [8, 9]. Despite the solved crystal structures, the theories that proposed to explain the higher reversibility of thermal denaturation of BLA are not clearly corroborated. And the only apparent feature is that BLA contains more ionic interactions than other less thermostable α-amylases [10].

To date, there have been numerous works done with respect to the reversibility of thermal denaturation of BLA [11–13]. Declerck et al. have demonstrated that the reversibility of thermal denaturation of BLA is determined by the regions of domain B. In addition, the regions between domain A and B also influence the reversibility of thermal denaturation of BLA [11]. In one report, removal of an amide-containing side chain in BLA by mutation of Asn190 to phenylalanine (N190F) dramatically improved the reversibility of thermal denaturation of the enzyme [3]. The crystal structure of BLA shows that Asn190 is contained within the 178–199 long loop region in domain B. Furthermore, this long loop in several bacterial α-amylase was focus of attempts to improve their reversibility of thermal denaturation [14–16]. When compared with BAA and BSTA, the major reason of improved reversibility of thermal denaturation of BLA is a short deletion in the turn following the fourth β-strand in this long loop. Some encouraging improvements in reversibility of thermal denaturation were detected by deleting an Arg-Gly sequence in the corresponding region [14, 17]. This long loop might play a key role in determining the overall stability of BLA, and a stabilizing effect of this long loop might help to enhance the reversibility of thermal denaturation of BLA.

In this study, the amino acid sequence of this long loop in BLA was used to identify five homologs in NCBI database, and the mutations of some un-conservative sites in this long loop and close proximity position were carried out. Seven mutants were generated and their reversibilities of thermal denaturation and catalytic efficiencies were compared with those of wild-type BLA, and the reasons for the improvements observed were rationalized in terms of their modelled structures.

## Materials and methods

### Bacterial strains, plasmids, and materials

The α-amylase gene (GenBank accession number AAA22240.1) from *Bacillus licheniformis* was synthesized by Sangon Biological Engineering Technology & Services Co. Ltd. (Shanghai, China). Plasmid pET-20b (+) (Novagen) was used for subcloning to generate recombinant plasmid pET-20b-amylase. PelB single peptide was used to make secretory expression of recombinant α-amylase. The strain *E*. *coli* JM109 (TakaRa, Dalian, China) was used for cloning work; The strain *E*. *coli* BL21 (DE3) (Novagen; Madison, WI, USA) was used for the expression of recombinant α-amylase.

DNA Ligation Kit, the MutanBEST Kit, polymerase chain reaction (PCR) reagents, restriction endonucleases, the PrimeSTAR HS DNA polymerase, and the Agarose Gel DNA Extraction Kit were purchased from TaKaRa (Dalian, China). Isopropyl β-D-1-thiogalactopyranoside and ampicillin were purchased from Sangon Biological Engineering Technology & Services Co. Ltd. All chemicals and reagents were of higher quality or analytical grade.

### Site-directed mutants

A multiple sequence alignment was constructed using ClustalW software [18]. The Swiss-Model (http://swissmodel.expasy.org/) protein-modeling server [19, 20] was used to prepare the homology models of the structures of the BLA and its mutants. The crystal structure of *B*. *licheniformis* alpha-amylase (PDB ID, 1OB0) was applied as a model [6]. Pymol software was used to visualize the structures. Overlap extension PCR was used to generate the mutations through site-directed mutagenesis [21]. The sequences of the oligonucleotides are listed in Table 1.

**Table 1:** Oligonucleotide primers used for site-directed mutagenesis.

Mutant	Nucleotide sequence (5’→3’)^a^
A269K	AGTAACGATCTGGGCGCT(AAA)CTGGAAAACTACCTG
S187D	TGGGACTGGGAGGTTGTT(GAC)AACGAGTTTGGTAACTAC
N188S	TGGGACTGGGAGGTTGTTAAC(ACC)GAGTTTGGTAACTAC
N188T	TGGGACTGGGAGGTTGTTAAC(TCT)GAGTTTGGTAACTAC
Y262F	TTCACGGTGGCCGAATAC(TTT)TGGAGTAACGATCTG
S186D/N187T	TGGGACTGGGAGGTTGTTAAC(GACACC)GAGTTTGGTAACTAC

^a^Nucleotides underlined correspond to the codons chosen for mutation. Nucleotides in parentheses replaced the underlined nucleotides.

### Expression of recombinant proteins

Lysogeny broth (LB) medium [17] was used for the seed culture. The modified terrific Broth (TB) medium [17] was employed in all shake-flask cultures. These media were supplemented with 0.1 g·L^-1^ ampicillin.

A frozen glycerol stock was used to inoculate in a 250 mL shake flask, which contained 50 mL of LB medium, to obtain a seed culture. The culture was grown at 37°C and 200 rpm for 8 hours. Then 2 mL the seed culture was inoculated into 50 mL TB medium, which was grown at 37°C in a rotary shaker (200 rpm). When the optical density (OD) at 600 nm reached 1.0, IPTG was added to induce the expression of the α-amylase, with a final concentration of 0.02 mM at 25°C. After cultivating the strains for 48 h, the culture medium, which contained the secreted α-amylase, was centrifuged at 13,800×*g* for 5 min at 4°C and then used directly for the protein purification.

### Purification of α-amylase

The recombinant α-amylase was secreted into supernatants. First, the supernatants were adjusted to pH 7.0 and then incubated at 80°C in the presence of 10 mM Ca^2+^ for 1 h. Plenty of the unnecessary proteins were denatured and precipitated during this step, while BLA still kept soluble and active [22]. The resulting supernatant was subjected to 30% (w/v) ammonium sulfate fractionation. The precipitate was collected by centrifugation at 13,800×*g* for 5 min at 4°C. Then the precipitate was suspended in pure water. The resulting clear solution was dialyzed against sodium phosphate buffer (pH 7.0, 20 mM). Then ÄKTA FPLC system was used in the anion exchange chromatography. A DEAE-Sepharose column (160 mm×10 mm; Pharmacia Biotech) pre-equilibrated with 20 mM sodium phosphate buffer (pH 7.0) was applied in this process. Finally, the adsorbed proteins were eluted by a linear gradient of 0 to 1 M NaCl in 20 mM sodium phosphate buffer (pH 7.0). The fractions containing α-amylase activity were pooled and dialyzed against 20 mM sodium phosphate buffer (pH 7.0) overnight at 4°C.

### Enzyme assays and kinetic parameters

Alpha-amylase activity was determined by measuring the formation of reducing sugar released during hydrolysis of 1% (w/v) soluble starch in 50 mM citrate buffer (pH 6.0) at 70°C for 5 min. The reaction mixture contained 1.0 mL soluble starch, 0.9 mL citrate buffer, and 0.1 mL diluted enzyme. The dinitrosalicylic acid method was used to measure the amount of reducing sugar [23]. One unit of α-amylase activity was defined as the amount of enzyme that released 1 μmol of reducing sugar per min under the assay conditions. A standard curve was constructed using glucose as a substrate.

The kinetic parameters (*K*_m_, *V*_max_, *k*_cat_ and *k*_cat_/*K*_m_) of BLA and its mutants were determined in 50 mM citrate buffer (pH 5.5) at 70°C using soluble starch as the substrate. The concentration of soluble starch was ranged from 1 to 20 g·L^-1^. GraphPad Prism software (GraphPad Software Inc., San Diego, CA) was used to estimate the *V*_max_ and *K*_m_ by fitting the initial rate data to the Michaelis-Menten equation. As the precision of *K*_m_ values is relatively low, the estimated standard deviation of *k*_cat_/*K*_m_ values is calculated according to the parameters of *k*_cat_.

### Effect of temperature and pH on enzyme activity

The effect of pH on α-amylase activity was determined by measuring its activity in the pH range from 4.5 to 7.5 at 70°C. The kind of buffer used in this part was phosphate buffer (pH 4.5 to 7.5, 50 mM). The effect of temperature on α-amylase activity was measured between 60 and 95°C. Before the beginning of enzyme assays, the phosphate buffer (50 mM, pH 6.0) and substrate (1% soluble starch) were pre-incubated at each temperature for 5 min.

### Reversibility of thermal denaturation

Samples with a protein concentration of 0.2 mg·mL^-1^ at 20 mM citrate buffer (pH 5.0) containing 10 mM Ca^2+^ were incubated at 95°C to test the reversibility of thermal denaturation of enzymes. The residual activity was detected using the method described in Section 2.5 after thermal treatment.

### Protein analysis and SDS-PAGE

The Bradford method was used to measure protein concentrations with bovine serum albumin as the standard [24]. The molecular weight of α-amylase was determined using sodium dodecyl sulfate-polyacrylamide gel electrophoresis (SDS-PAGE) analysis [25].

## Results and discussion

### Selection of mutation sites

Previously, the results of several reports demonstrated that some amino acids in the 178–199 loop region (BLA numbering) in domain B plays a significant effect in reversibility of thermal denaturation of α-amylases [14–16]. The deletion of two amino acids between Gly179 and Lys180, in the corresponding region of other α-amylases, has been proved beneficial to their reversibilities of thermal denaturation [15, 16]. In addition, mutation at site of 190 has been investigated as well [11]. So, N190F mutation was initiated before the beginning of investigation of this loop. In present study, the selection of mutation sites began with an alignment of the amino acid sequence of the long loop (Phe178–Pro199) in BLA with that of five homologs in the NCBI database using ClustalW software (S1 Fig in the supplemental material). As shown in S1 Fig, there were seven un-conservative sites in the long loop. Three sites (Gly179, Lys180, and Asn190) has been investigated previously. In this study, in order to investigate the function of the four remaining sites (Gln178, Ala181, Ser187, Asn188) on the reversibility of thermal denaturation of BLA, they were chosen as potential mutations for further analysis.

**Fig 1:**
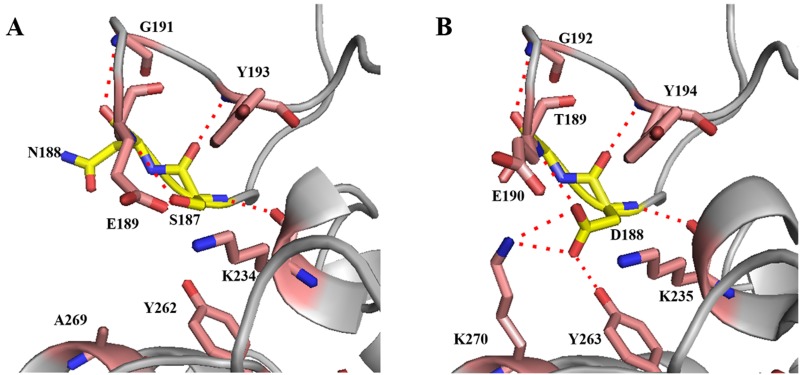
Close-up view of the structural differences between BLA (from PDB ID: 1OB0) and BSTA (from PDB ID: 4UZU) around the area of the loop. Cartoon representations of the structures are rendered in gray. **(A) Close-up view of the structure around Ser187 and Asn188 in BLA (C, yellow; N, blue; O, red). (B) Close-up view of the structure around Asp188 and Thr189 in BSTA (C, yellow; N, blue; O, red).** The positions of relative residues around the two key sites are shown in sticks as well (C, magenta; N, blue; O, red). The intermolecular forces are indicated as red stippled lines.

The PoPMuSiC algorithm is an online tool that predicts the changes in thermodynamic stability caused by single-site mutations [26]. And these four sites were subsequently replaced by the residues seen at that position in the alignment. Five mutants (N178R, A181D, S187D, N188T, N188S) were initiated to evaluate the stability changes using PoPMuSiC algorithm. Among these mutations, only mutation on Asn188 was predicated to have stabilizing effect, and the ΔΔG values of N188T and N188S predicated using PoPMuSiC algorithm were -0.30 and -0.06 kcal/mol. The N188T and N188S mutations might improve the reversibility of thermal denaturation of BLA. For the adjacent space between Ser187 and Asn188, and potential synergistic effects might exist between these two sites, the mutation on Ser187 was also investigated. In addition, three-dimensional structures comparison between BLA (1OB0) and BSTA (PDB ID: 4UZU) showed that there are more intermolecular forces in the corresponding region around Asp188 and Thr189 in BSTA compared with that of BLA (Fig 1). In particular, Asp188 introduces a hydrogen bond to Tyr263 and a salt bridge to Lys270 (Fig 1B). These intermolecular forces might help to stabilize the loop where Asp188 and Thr189 located. The mutation of corresponding region in BLA (A269K) might have the similar effects, because the structure and amino acid composition of these two enzymes are quite similar.

### Construction, expression and purification of mutant enzyme

To test the hypotheses described above, the single mutants S187D, N188S, N188T, A269K were created. To investigate the potential interaction effects of these single mutations on reversibility of thermal denaturation, the double mutation S187D/N188T, A269K/S187D and the triple mutation A269K/S187D/N188T were constructed in expression plasmids and expressed into the culture medium using *E*. *coli* BL21(DE3). The recombinant α-amylases, which were purified from the culture supernatants using ion-exchange chromatography, migrated through SDS-PAGE gels with apparent molecular masses of approximately 53 kDa. When assayed using 1% soluble starch as the substrate, S187D/N188T, A269K/S187D and A269K/S187D/N188T displayed specific activities 19.8%, 30.1% and 26.4% greater than that of the wild-type enzyme, respectively, whereas the specific activity of the A269K mutant was 35.8% lower than that of the wild-type (S1 Table). Furthermore, the specific activity of N188S, S187D, and N188T mutant displayed similar values of specific activity to the wild-type enzyme.

### Effects of pH and temperature on enzyme activity

**Fig 2:**
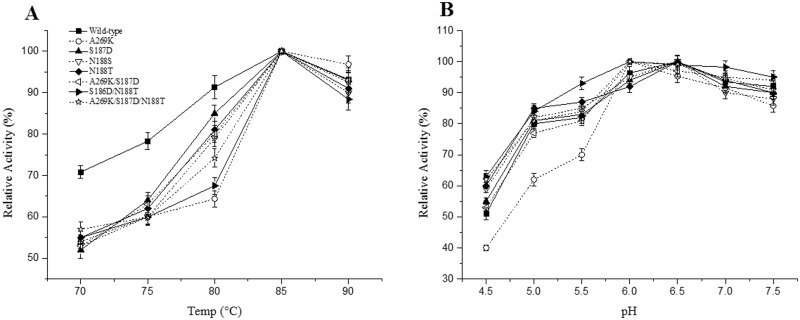
Effects of pH and temperature on the activities of BLA and its mutants. **(A) Optimal temperatures of BLA and its mutants.** Activities were measured in 50 mM phosphate buffer (pH 6.0) at 70 to 90°C for 5 min. The activity of BLA at 85°C was defined as 100%. **(B) Optimal pH values of BLA and its mutants.** Assays were carried at 70°C for 5 min in buffers of various pH. The highest activity of enzyme at each pH was defined as 100%. The activities are expressed as percentages. The data represent the average of three independent measurements. The error bars represent the standard deviation.

The α-amylase used in the industrial degradation process is required to be active at high temperature and under acidic condition [27]. In the present study, the temperature-dependence of the enzyme activities was measured from 70 to 90°C using soluble starch as the substrate in phosphate buffer (pH 6.0). The results showed that the optimal temperature of all seven mutants was 85°C, which was similar to that of the wild-type enzyme (Fig 2A). The wild-type BLA and its mutants hydrolyze 1% soluble starch at various pH values in the range of 4.5 to 7.5 at 70°C. The results showed that the pH optimum of the wild-type enzyme and N188S, N188T, S187D mutants was 6.5, whereas the mutants A269K, A269K/S187D, and A269K/S187D/N188T showed pH optima at pH 6.0. In addition, the mutant S187D/N188T showed the optimal pH of 5.5 (Fig 2B).

### Thermal denaturation assays

**Fig 3:**
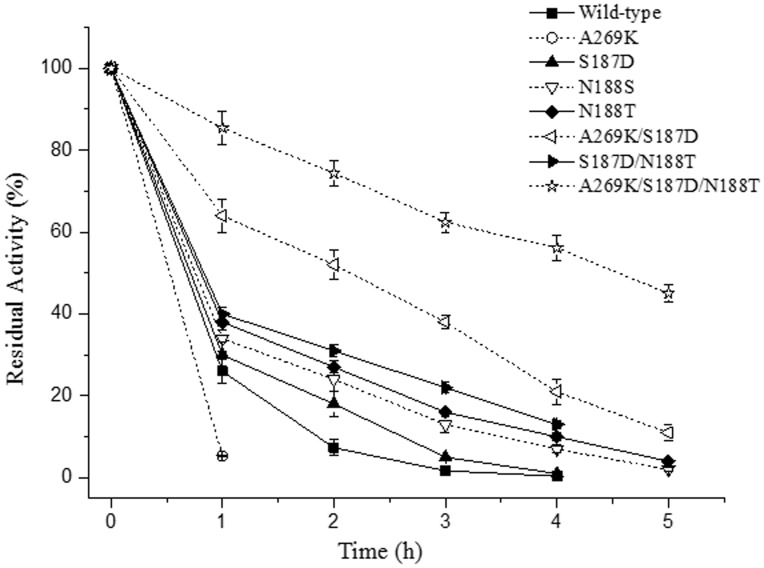
Thermal denaturation of BLA and its mutants in the presence of 10 mM Ca^2+^ at pH 5.5 at 95°C. The data represent the average of three independent measurements. The error bars represent the standard deviation.

To evaluate the reversibilities of thermal denaturation of the wild-type enzyme and its mutants, the enzymes were incubated at 95°C and pH 5.5 in the presence of 10 mM Ca^2+^, and the residual activity was measured at 70°C after different incubation times. As shown in Fig 3, the activity of single mutant A269K decreased drastically. Only about 5% residual activity could be detected after treatment at 95°C for 1 h. It is confirmed to the previous study reported by Declerck *et al*. [3]. The mutation S187D, N188T and N188S had slightly beneficial to reversibility of thermal denaturation. In addition to the single mutations, our study also examined the possibility of combining single mutations to obtain increased effects. The S187D/N188T double mutant was substantially more thermostable than the wild-type enzyme and its individual single mutations. Despite the negative effect on reversibility of thermal denaturation of A269K mutation, the A269K/S187D mutant had satisfactory reversibility of thermal denaturation, which displayed a half-life of about 120 min. Nevertheless, the stability of A269K/S187D mutant can be further improved by N188T mutation. The half-life of A269K/S187D/N188T was raised to 270 min, which was 9-fold greater than that of the wild-type enzyme.

**Fig 4:**
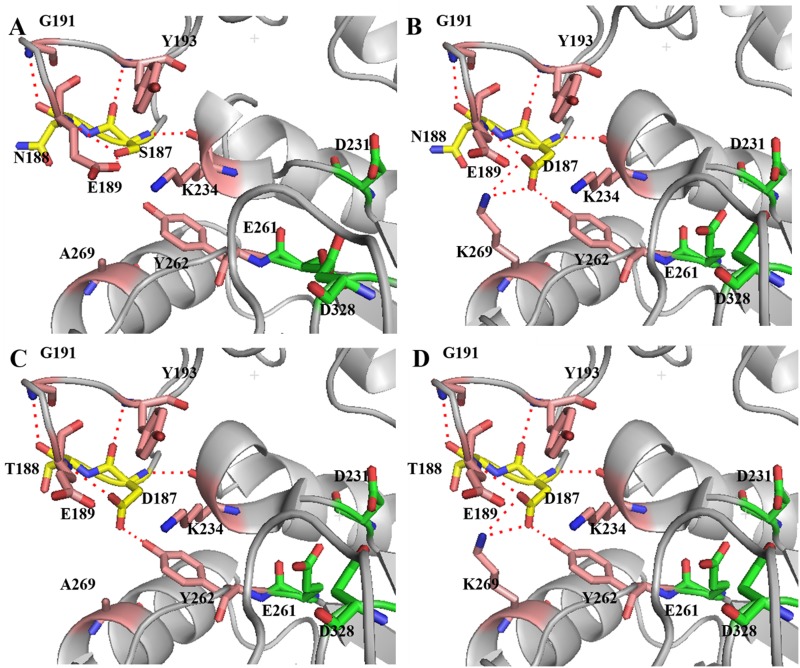
Close-up views of the modelled structural changes introduced by mutation at Ser187, Asn 188 and Ala269. **(A) Wild-type enzyme; (B) A269K/S187D mutant; (C) S187D/N188T mutant; D) A269K/S187D/N188T mutant.** The three catalytic residues (C, green; N, blue; O, red), and the residues at sites of mutation (C, yellow; N, blue; O, red) are shown as sticks. The positions of relative residues around the mutation sites are shown in sticks as well (C, magenta; N, blue; O, red). The hydrogen bonds are indicated as red stippled lines. The figure was produced using PyMol.

To explain the differences in reversibility of thermal denaturation in terms of structure, three-dimensional models of the BLA and its mutants were constructed based on the crystal structure of the α-amylase from *Bacillus licheniformis* (PDB ID, 1OB0), with which the BLA used in this study shares 99% sequence identity (S2 Fig in the supplemental material), using the SwissModel server. In wild-type BLA (Fig 4A), Ser187 and Asn188 are located at a long loop and involved in a hydrogen bonding network primarily within domain B. Meanwhile, the hydrogen bond between the main chains of Ser187 and Lys234 helps immobilize the loop in which Ser187 and Asn188 are located.

The A269K/S187D double mutation has obvious influence on the reversibility of thermal denaturation of enzyme. As shown from three-dimensional model in Fig 4B, compared with the wild-type enzyme, the double mutants A269K/S187D had more intramolecular interactions around Asp187. Mutation of Ala269 to lysine would introduce salt bridges between Lys269 and Asp187. Furthermore, another new hydrogen bond is generated between Asp187 and Tyr262 as well. These changes in the intermolecular forces pattern might have a positive effect on the immobilization of this long loop. In a previous report, replacement of Ala269 by lysine reduced reversibility of thermal denaturation of BLA [3]. The explanation of this phenomenon is that introduction of lysine at position 269 unfavorably modifies electrostatic interactions in the region where Lys234 and Glu189 located. In the present study, the single mutation A269K displayed negative effect on reversibility of thermal denaturation of BLA as well. However, when mutation A269K is combined with S186D, the reversibility of thermal denaturation of BLA is enhanced dramatically.

In the double mutant S187D/N188T, the hydrogen bond between the side chains of Asp187 and Glu189 is disappeared. Instead, a new hydrogen bond is generated between the side chains of Tyr262 and Asp187 (Fig 4C), like the one present in BSTA. The new hydrogen bond might stabilize the long loop as well. Furthermore, deamidation of Asn residues has emerged as an important cause of thermal inactivation of BLA [28]. Removal of the amide-containing side chain at Asn188 by site-directed mutagenesis to form N188T might weaken the effect of Asn deamidation, with consequent change in conformation of this loop, which would likely impact negatively on nearby residues on this loop that form part of the active site of this enzyme. When the single mutations combined together (A269K/S187D/N188T), the reversibility of thermal denaturation of enzyme can be further improved. To further verify the hypotheses that the improved reversibility of thermal denaturation of enzyme is caused by the stabilizing effect of mutations to the loop, another mutation (Y262F) was initiated in this study. The result showed that the reversibility of thermal denaturation of A269K/S187D/N188T/Y262F mutant was decreased compared with A269K/S187D/N188T mutant (S3 Fig).

### Kinetic parameters of the wild-type and mutant enzyme

A comparison of the kinetic parameters of the wild-type enzyme with those of seven mutants at pH 5.5 was carried out using soluble starch as the substrate. The kinetic behavior of all of these enzymes was determined by fitting the data with the Michaelis-Menten equation. As shown in Table 2, the *K*_m_ values of A269K, S187D, N188S, N188T, and S187D/N188T mutants were similar to the wild-type enzyme. However, A269K/S187D and A269K/S187D/N188T mutants have *K*_m_ values approximately 25% less than that of the wild-type enzyme. A modelled structural analysis shows that the mutations of A269K and S187D would introduce salt bridges between Lys269 and Asp187and a new hydrogen bond between Asp187 and Thy262 (Fig 4B). These intramolecular interactions might slightly influence the spatial structure of the loop where Glu261 located, which involved in the bonding of the substrate to the active site of enzyme [29]. This might create a more favourable environment for substrate-bonding compared to the wild-type enzyme. When the mutation of A269K or S187D was initiated individually, the intramolecular interactions might not strong enough to affect the position, which has influence on substrate-bonding. In addition, the catalytic constants of A269K decreased by 39.8%. Compared with wild-type enzyme, the single mutants S187D, N188S, and N188T displayed similar *k*_cat_ values. Among the mutants of increased catalytic constants, the A269K/S187D and A269K/S187D/N188T showed similar increase in *k*_cat_ values, which was approximately 30% more than that of wild-type enzyme. Moreover, compared to that of the wild-type enzyme, A269K mutant displayed decreased catalytic efficiency. Compared with wild-type enzyme, the single mutants S187D, N188S, and N188T displayed similar catalytic efficiency. And the rest of mutants showed enhanced catalytic efficiency. The A269K/S187D/N188T mutant displayed highest *k*_cat_/ *K*_m_ value, which was 1.84-fold that of the wild-type enzyme. As the differences in substrates and conditions used in assays, it is difficult to compare the kinetic parameters of BLA with other α-amylases. The only one that could be compared is the α-amylase from *Bacillus stearothermophilus*, which has been investigated in our previous report using the same substrate and condition in enzyme assays [17]. The catalytic efficiency of α-amylase from *Bacillus stearothermophilus* is higher than that of BLA. However, the better reversibility of thermal denaturation and acidic stability of this mutant BLA makes it more suitable for starch liquefaction at high temperature and under acidic condition.

**Table 2:** Kinetic parameters of wild-type and mutant BLA at pH 5.5 and 70°C.

Enzyme	*k*_cat_ (×10^3^ s^-1^)	*K*_m_ (g·L^-1^)	*k*_cat_/ *K*_m_ (×10^3^ g·L^-1^·s^-1^)
Wild-type	5.40±0.17	1.7±0.1	3.18±0.09
A269K	3.25±0.16	1.7±0.1	1.91±0.05
S187D	5.42±0.17	1.7±0.1	3.19±0.09
N188S	5.81±0.18	1.8±0.1	3.23±0.10
N188T	5.83±0.19	1.8±0.1	3.24±0.10
A269K/S187D	7.12±0.21	1.3±0.1	5.48±0.17
S187D/N188T	5.89±0.17	1.6±0.1	3.68±0.17
A269K/S187D/N188T	7.04±0.21	1.2±0.1	5.87±0.17

The reversibility of thermal denaturation of BLA has been improved by rational protein engineering techniques in many previous reports [11–13]. Recently, the tolerance of BLA toward low pH was enhanced by site-directed mutagenesis [30] and directed evolution [31]. Substantial effort has been devoted to identifying the determinants of the pH-rate profile of BLA [32, 33]. Comparatively little work has been done with respect to combined improved catalytic efficiency and reversibility of thermal denaturation. In one study, Liu et al. [30] studied the catalytic efficiency of BLA using 4, 6-ethylidene(G7)-p-nitrophenyl(G1)-α, d-maltoheptaoside (Et-G7-PNP) as the substrate. Liu et al. [30] reported that the mutant L134R/S320A had an approximately 14-fold improvement in catalytic efficiency of BLA at pH 4.5. However, there are no significant differences in catalytic efficiency at pH 5.5 and 6.5 between L134R/S320A and the wild-type enzyme [30]. Despite its improved catalytic efficiency at pH 4.5, the wild-type BLA and its mutants showed similar thermal inactivation behavior, indicating that there was no change in the reversibility of thermal denaturation. In this study, the reversibility of thermal denaturation of mutant BLA was improved with a half-life of 270 min at pH 5.5 and 95°C, a 9-fold improvement in half-life compared with that of wild-type enzyme. The better reversibility of thermal denaturation and catalytic efficiency of this mutant BLA make it more suitable for starch liquefaction at high temperature and under acidic condition. The mutant enzymes, particularly A269K/S187D/N188T, are potentially useful for application in the starch industry. The engineering work in this study provides a good example of the extent to which an enzyme can be remodeled in order to improve its natural performance and fulfill industrial requirements.

## Conclusions

The catalytic efficiency and reversibility of thermal denaturation of wild-type BLA were improved through site-directed mutagenesis. The most thermostable mutant protein, A269K/S187D/N188T, exhibited a 9-fold improvement in half-life at 95°C and pH 5.5, compared with the wild-type enzyme. The improvement in reversibility of thermal denaturation is likely to be a consequence of the immobilization of the loop containing Ser187 and Asn188. The catalytic efficiency of mutant A269K/S187D/N188T was improved as well. The mutant enzymes, particularly A269K/S187D/N188T are potentially useful for application in the starch industry.

## Supporting information

S1 Table Specific activities of BLA and its mutants using 1% soluble starch as the substrate. (DOCX)

S1 Fig Comparison of amino acid sequences of the long loop in BLA and five homologs^a^. ^a^The accession number of *Geobacillus Stearothermophilus* α-amylase in NCBI is WP_050582898; The accession number of *Bacillus glycinifermentans* α-amylase in NCBI is WP_046130478; The accession number of *Risungbinella massiliensis* α-amylase in NCBI is WP_044641732; The accession number of *Bacillus amyloliquefaciens* α-amylase in NCBI is WP_065521485; The accession number of *Bacillus megaterium* α-amylase in NCBI is AMX23350. The long loop is indicated by using black rectangle.
(TIF)

S2 Fig Comparison of amino acid sequences of BLA with the α-amylase of which the crystal structure has been investigated. The differences of amino acid between the two α-amylases are indicated by red.
(TIF)

S3 Fig Thermal denaturation of A269K/S187D/N188T/Y262F mutant and A269K/S187D/N188T mutant in the preference of 10 mM Ca^2+^ at pH 5.5 at 95°C. (TIF)
