# Supplementary material for: Improving the reversibility of thermal denaturation and catalytic efficiency of Bacillus licheniformis α-amylase through stabilizing a long loop in domain B
Source: PLoS One. 2017 Mar 2;12(3):e0173187. doi: 10.1371/journal.pone.0173187 (PMC5333897; doi:10.1371/journal.pone.0173187)
Supplement: S1 Table — (DOCX) [file pone.0173187.s001.docx]

| Enzyme | Specific Activity (U·mg^-1^) |
| --- | --- |
| Wild-type | 5794±132 |
| A269K | 3718±115 |
| S187D | 5781±135 |
| N188T | 5850±138 |
| N188S | 5820±130 |
| A269K/S187D | 7590±168 |
| S187D/N188T | 6944±171 |
| A269K/S187D/N188T | 7323±162 |
