# Supplementary figures and images for: Improving the reversibility of thermal denaturation and catalytic efficiency of Bacillus licheniformis α-amylase through stabilizing a long loop in domain B
Source: PLoS One. 2017 Mar 2;12(3):e0173187. doi: 10.1371/journal.pone.0173187 (PMC5333897; doi:10.1371/journal.pone.0173187)

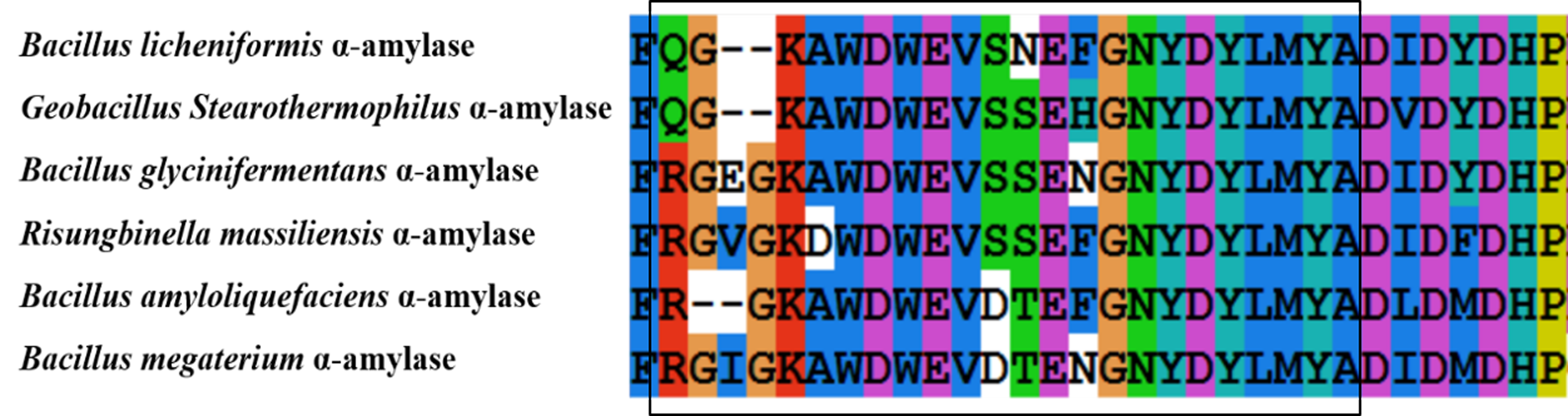

Supplement: S1 Fig — aThe accession number of Geobacillus Stearothermophilus α-amylase in NCBI is WP_050582898; The accession number of Bacillus glycinifermentans α-amylase in NCBI is WP_046130478; The accession number of Risungbinella massiliensis α-amylase in NCBI is WP_044641732; The accession number of Bacillus amyloliquefaciens α-amylase in NCBI is WP_065521485; The accession number of Bacillus megaterium α-amylase in NCBI is AMX23350. The long loop is indicated by using black rectangle. (TIF) [file pone.0173187.s002.tif]

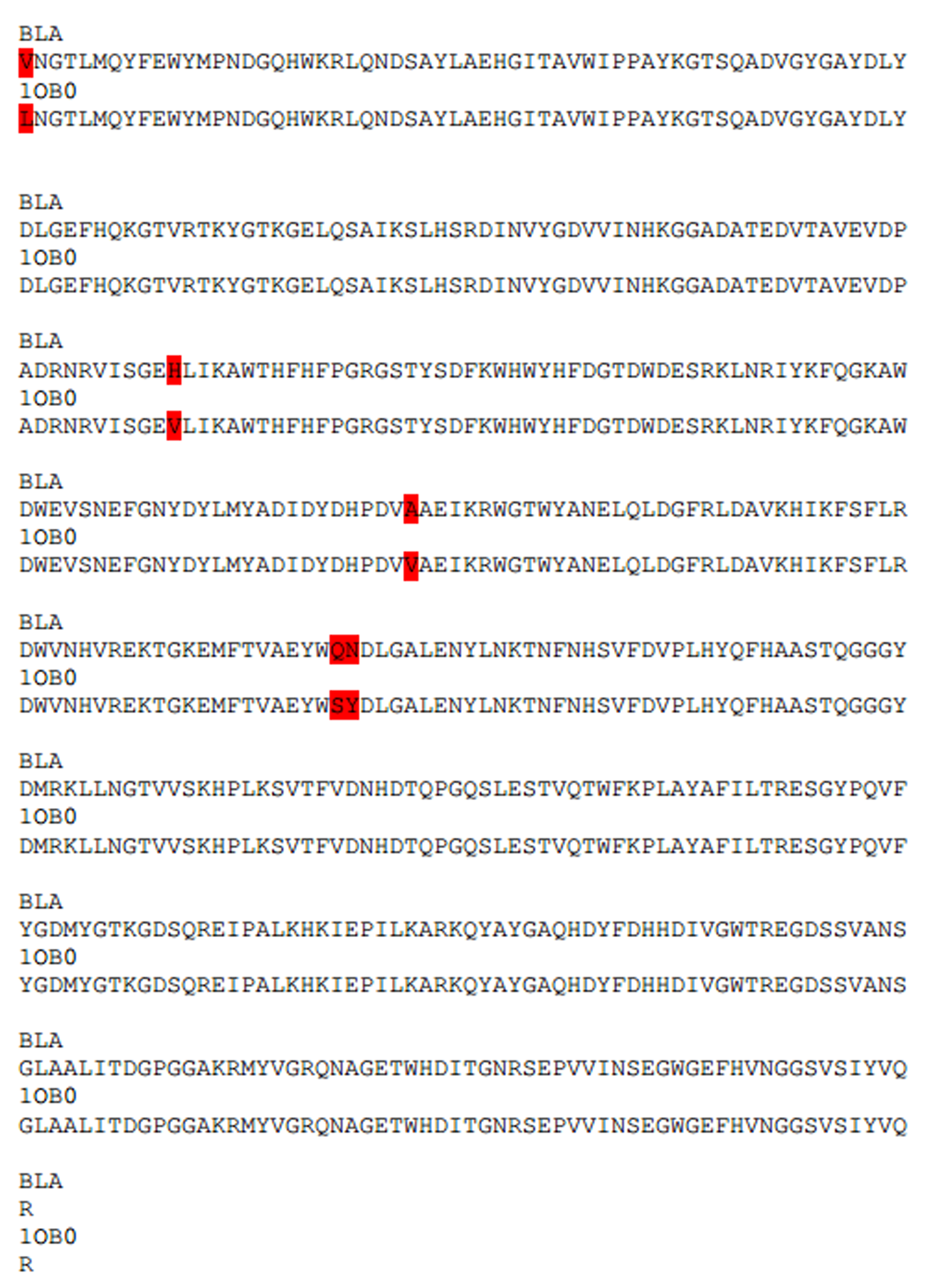

Supplement: S2 Fig — The differences of amino acid between the two α-amylases are indicated by red. (TIF) [file pone.0173187.s003.tif]

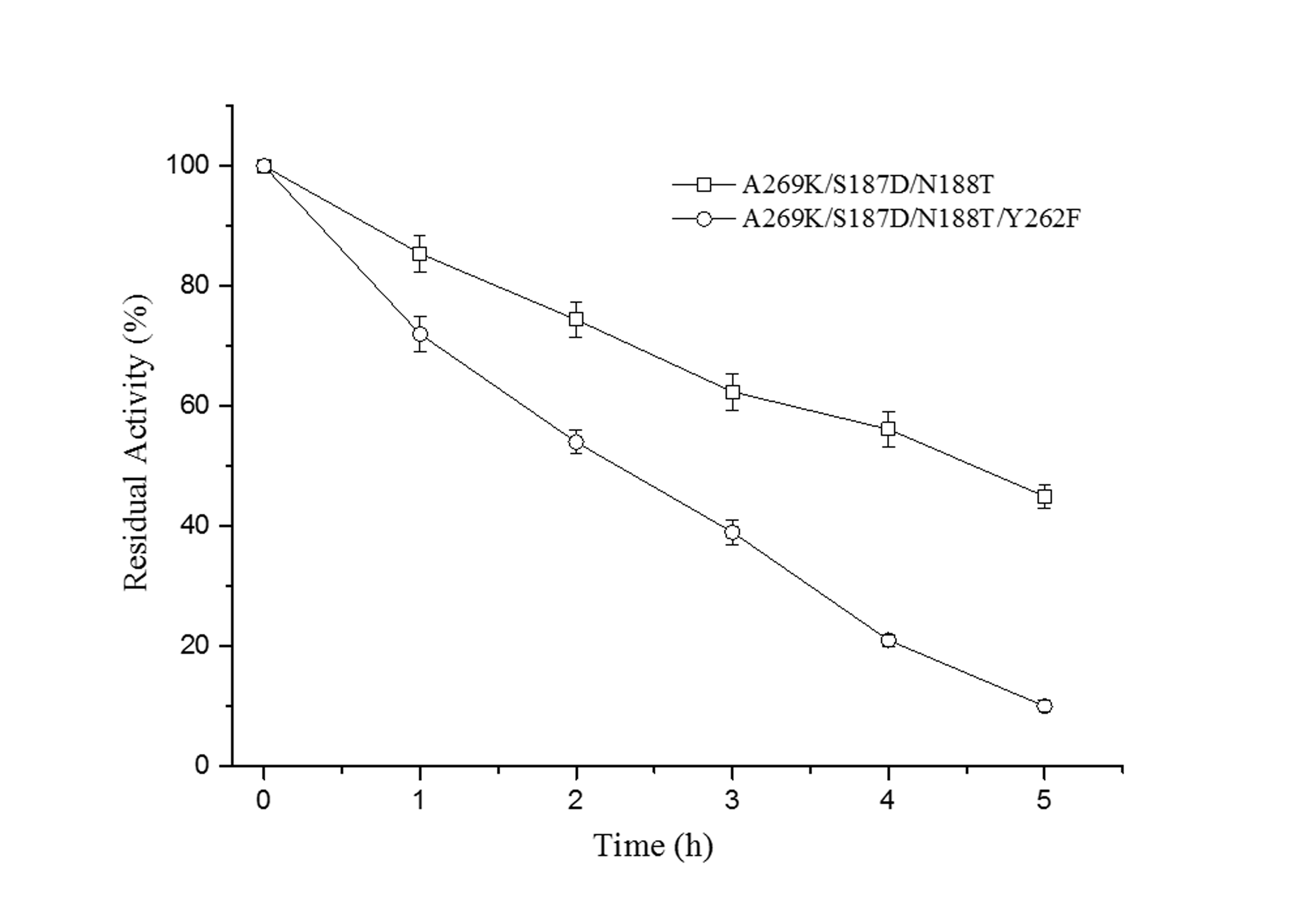

Supplement: S3 Fig — (TIF) [file pone.0173187.s004.tif]
